# Age-related changes in olfactory function: electrophysiological recordings from the olfactory mucosa and human brain

**DOI:** 10.1038/s41514-026-00478-3

**Published:** 2026-09-17

**Authors:** Yiling Mai, Henriette Zimmeck, Cagdas Guducu, Thomas Hummel

**Affiliations:** 1 Smell and Taste Clinic, Department of Otorhinolaryngology, Faculty of Medicine Carl Gustav Carus, Technische Universität, Dresden, Germany; 2 Dokuz Eylul University Faculty of Medicine Department of Biophysics, 35340 Balcova, Izmir Turkey; 3 Department of Nutritional Science & Food Management, Ewha Womans University, Seoul, Republic of Korea

**Keywords:** Neuroscience, Physiology

## Abstract

Age-related olfactory decline in humans is well-documented, yet the underlying peripheral neural mechanisms remain poorly understood. We investigated age-related olfactory decline across psychophysical, nasal mucosal, and central neural levels in 73 healthy adults across the lifespan. Participants underwent Sniffin’ Sticks testing and simultaneous recordings of mucosal electro-olfactograms (EOG) and cerebral chemosensory event-related potentials (CERP) in response to olfactory and trigeminal stimulation. Older adults exhibited significantly lower psychophysical olfactory scores, prolonged CERP P2 latencies, and reduced spectral power across theta, delta, and beta bands at peripheral (EOG) and cerebral (Fz, Cz, and Pz) sites. These findings demonstrate a systemic decline of olfactory function. Although the olfactory mucosa remains functionally active, diminished neural recruitment and oscillatory power, coupled with slower central processing, characterize olfactory aging. These objective electrophysiological markers provide a comprehensive profile of sensory decline in the aging population.

## Introduction

The sense of smell allows us to perceive a wide range of odors, playing a crucial role in environmental hazard avoidance^1^, shaping our eating behavior^2,3^, facilitating social communication^4,5^, and even modulating our emotions^6^, memories^7^, and cognition^8^. Consequently, it is not surprising that olfactory loss can significantly impact quality of life^9,10^, well-being^11^, while also predict neurodegenerative progression^12,13^ and higher mortality risk^14,15^. This profound link suggests that olfactory dysfunction is not merely a sensory deficit, but - much like the visual and auditory impairments, though less extensively studied - a critical physiological indicator of biological aging and systemic health.

Aging is the most prominent risk factor for olfactory loss. Psychophysical tests consistently observe decreased olfactory sensitivity, discrimination, and identification in older individuals^15–17^. Approximately 50% of individuals aged 65-80 years, and approximately 75–80% of those beyond 80 years, experience some degree of olfactory loss^15,16,18^. A meta-analysis of 175,073 participants also confirmed that olfactory dysfunction becomes more prevalent with age^19^. Beyond its sensory function, olfaction appears to be closely linked to the overall health of aging individuals, suggesting that it could serve as an early biomarker of age-related decline^15,20^. The inevitable process of normal aging, combined with the significant consequences of olfactory loss, underscores the clinical importance of investigating the mechanisms of age-related olfactory dysfunction.

Age-related olfactory dysfunction is considered to arise from multiple mechanisms, including degeneration of the olfactory epithelium, altered receptor function, reduction in mucosal metabolizing enzymes, changes in olfactory bulb volume or circuitry, and impairments in cortical processing^21,22^. However, evidence supporting these mechanisms has been derived from animal models, postmortem human tissue, or indirect behavioral assessments. Consequently, in human participants, how age-related changes at different levels of the olfactory system jointly affect the neural representation remains poorly understood.

Electrophysiological recordings provide insight into olfactory processing and reveal how aging affects the neural representation and interpretation of odors^23–25^. This includes EEG-derived chemosensory event-related potentials (CERPs) recorded from the scalp representing the central level^25,26^, and electro-olfactograms (EOG) recorded from the olfactory mucosa representing peripheral activity^23,27^. Beyond representing different processing levels, these two measures also differ in their underlying signal characteristics. The CERP is characterized by a small initial positive peak at around 250–320 ms, followed by a major negative peak at approximately 320–450 ms and a later positive component at  approximately 530–800 ms^28^. The EOG, in contrast, is a local-field potential, typically comprising an initial fast and brief positive deflection within the first 500 ms, followed by a large, slow negative peak, reflecting the depolarization of the olfactory sensory neurons, that often occurs at approximately 1–2 s, and a subsequent gradual return into a positive deflection that can occur as late as about 4 s^23,27,29^. Recording both, therefore, provides complementary, level-specific windows on how aging affects olfactory function from the periphery to the cortex.

Some studies have noted age-related alterations in the time domain of CERPs, with older individuals typically exhibiting longer ERP latencies and smaller amplitudes^24–26,30,31^. Several EEG studies^32–34^ have demonstrated that odor stimulation modulates spectral power across EEG-frequency bands (e.g., changes in theta and beta power with olfactory perception), supporting the use of frequency-domain metrics in CERP. However, EEG frequency-domain analysis has not yet been conducted to study age-related olfactory loss.

Unlike EEG, most EOG studies on aging rely on animal models, showing that aging affects peripheral olfactory encoding, demonstrating reduced EOG amplitudes in aged rodents, accompanied by epithelial degeneration and loss of olfactory sensory neurons^35^. However, direct studies on the effects of age on human EOG are lacking. Bridging this gap is crucial, as human olfactory mucosa represents the primary interface between the external environment and the central nervous system, and any age-related alterations at this initial stage would inevitably affect subsequent neural activity.

To address these gaps, we performed simultaneous EOG and CERP recordings to investigate how aging affects olfactory processing at both the mucosa and scalp levels during odor exposure. Specifically, we seek to answer the following questions: (1) Do EOG morphologies differ between young and old age groups? (2) How are age-related changes reflected in the frequency domain at both the peripheral (EOG) and central (CERP) levels of the olfactory system?

## Results

### Descriptive results

A total of 73 participants who passed the clinical screening (including a normal nasal endoscopy outcome) were initially included in the study, with 40 participants in the young group (22.6 ± 2.1 years, 25 female) and 33 participants in the old group (61.7 ± 7.8 years, 22 female). No significant sex difference was observed between young and old groups (χ^2^ = 0.14, *p* = 0.71). All 73 participants successfully completed the psychophysical assessments. Demographic information was shown in Table 1.

**Table 1:** Descriptive results

	Young (*n* = 40)	Old (*n* = 33)	*t*//*U*/χ^2^	*p*
	Mean ± SD/Median ± IQR	Mean ± SD /Median ± IQR		
**Sex (F: M)**	25:15	22:11	0.14	0.71
**Age**	22.55 ± 2.08	61.67 ± 7.84	27.88	<0.01
**BMI**	22.15 ± 2.63	24.20 ± 4.16	2.45	0.02
**Smoke (yes: no)**	2:38	3:30	0.47	0.49
**OT**	8.83 ± 2.42	7.48 ± 2.07	2.51	0.01
**OD**	14.00 ± 2.00	13.00 ± 2.00	314.5	<0.01
**OI**	14.00 ± 1.75	13.00 ± 2.00	487.5	0.049
**TDI**	36.00 ± 4.81	33.00 ± 3.63	334.5	<0.01

Group comparison between the young (<30 years) and old (>50 years) groups were performed using independent-samples *t*-tests for age, BMI, and OT; Mann–Whitney *U*-tests for OD, OI, and TDI; and Chi-square test (χ^2^) for sex and smoking status.

*F* female, *M* male, *BMI* body mass index, *OT* Sniffin’ Sticks odor threshold score (higher score, better sensitivity), *OD* Sniffin’ Sticks odor discrimination score, *OI* Sniffin’ Sticks odor identification score, *SD* standard deviation, *IQR* interquartile range.

During the electrophysiological recording session, five participants were excluded due to technical difficulties (e.g., broken electrodes or intolerance to the nasal electrode placement), leaving 68 participants who completed the electrophysiological recordings. Again, no significant sex difference was observed between young and old groups (χ^2^ = 0.50, *p* = 0.48). Following data processing, the number of participants with sufficient artifact-free epochs for CERP averaging was 62 for PEA, 62 for H_2_S, and 45 for CO_2_. For EOG averaging, the sample sizes were 59 for PEA, 57 for H_2_S, and 47 for CO_2_ (Supplementary Tables S1–S6). The accepted number of artifact-free epochs did not differ significantly between the young and the old groups across odor conditions (*t* = 0.43–1.27, *p’s* = 0.21–0.67).

### Psychophysical results

**Fig. 1:**
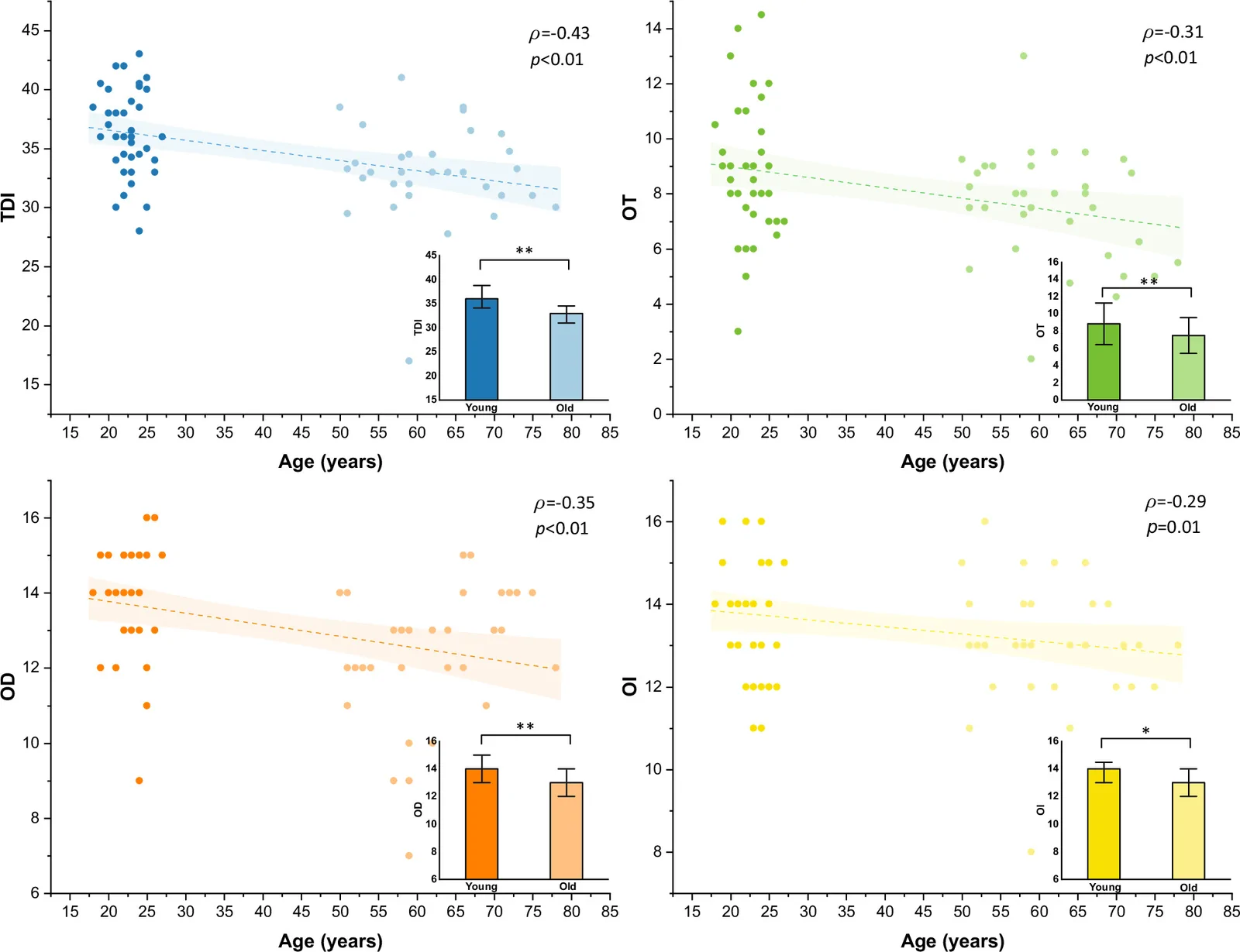
Relationship between age and psychophysiological olfactory test. Scatter plots visualize the correlations between age and olfactory measures, depicting individual data points, a linear fit (dashed line), and 95% confidence intervals (shaded areas) to illustrate age-related trends. Spearman coefficients (*ρ*) are reported, as age was not normally distributed in the full sample. Inset bar graphs display group-level average performance and variance. Depending on the distribution, data were presented as mean and standard deviation (OT) or median and interquartile range (OD, OI, and TDI). Group differences were evaluated using independent-samples *t*-tests (OT) or Mann–Whitney *U*-test (OD, OI, and TDI). *p < 0.05; **p < 0.01; TDI Sniffin’ Sticks total score, OT odor threshold test, OD odor discrimination test, OI odor identification test, Young: <30 years; Old: >50 years.

The old group consistently and significantly had lower OT (old vs. young: 7.48 ± 2.07 vs. 8.83 ± 2.42; *t* = 2.51, *p* = 0.01), OD (13.00 ± 2.00 vs. 14.00 ± 2.00; *U* = 314.50, *p* < 0.01), OI (13.00 ± 2.00 vs. 14.00 ± 1.75; *U* = 487.50, *p* = 0.049), and TDI (33.00 ± 3.63 vs. 36.00 ± 4.81; *U* = 334.50, *p* < 0.01) scores compared to the young group. Additionally, older age was significantly correlated with lower TDI, OT, OD, and OI scores (*ρ *= -0.43 to -0.29, *p’s* ≤ 0.01). See Fig. 1.

### Electrophysiological: time-domain results

**Fig. 2:**
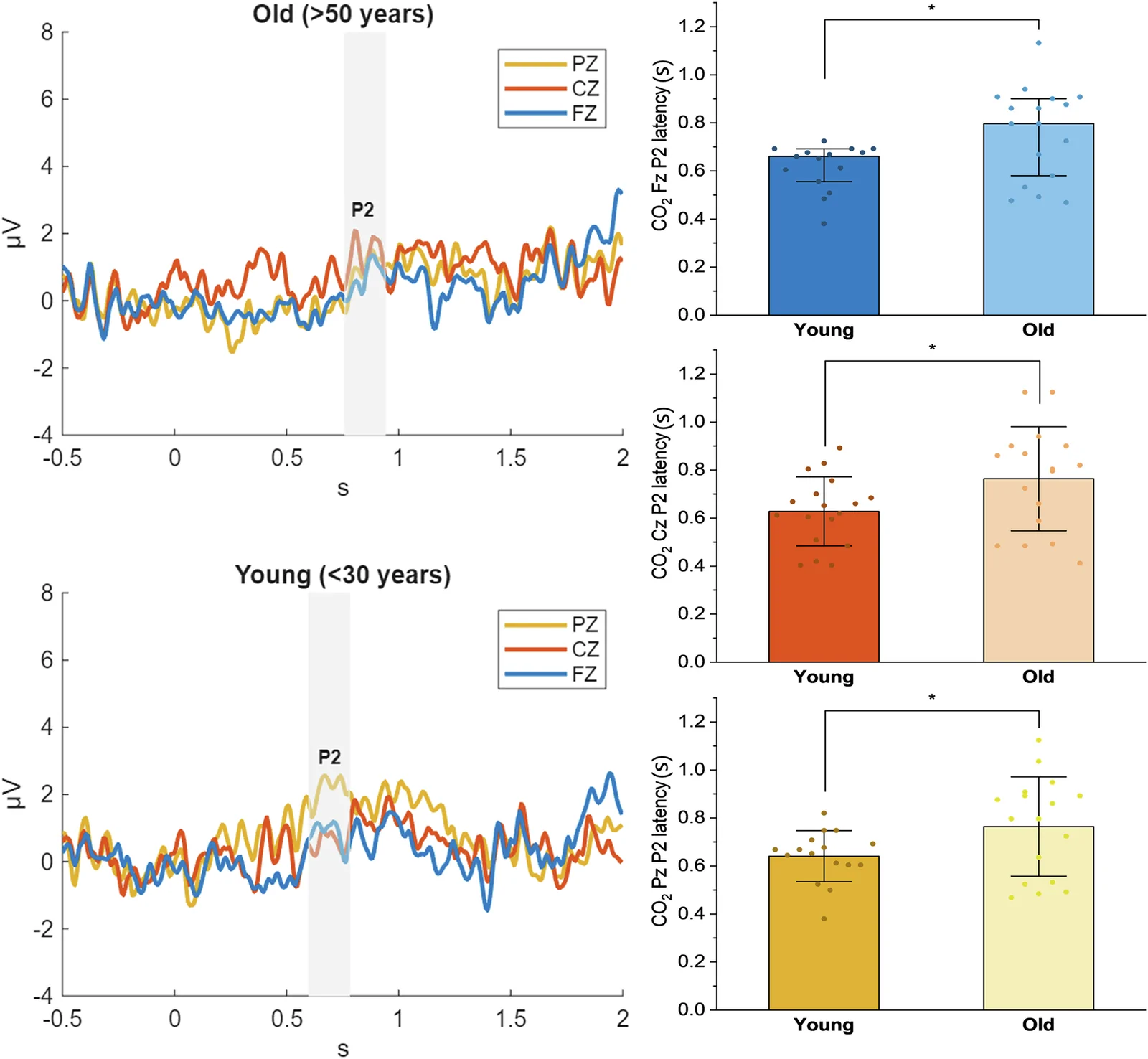
ERP Waveforms in CO_2_ condition in Old and Young Participants. Grand-average waveforms at Pz, Cz, and Fz electrode sites in old (>50 years) and young (<30 years) participants under the CO_2_ condition. The shaded region indicates the approximate time window of the P2 component. Inset bar plots with individual data points depict average group-level performance with variance. Depending on the data distribution, data are presented as the mean and standard deviation (Cz and Pz latency) or the median and interquartile range (Fz latency). Group differences were evaluated using independent-samples *t*-tests (Cz and Pz) or Mann–Whitney *U*-test (Fz). *p < 0.05; **p < 0.01.

**Fig. 3:**
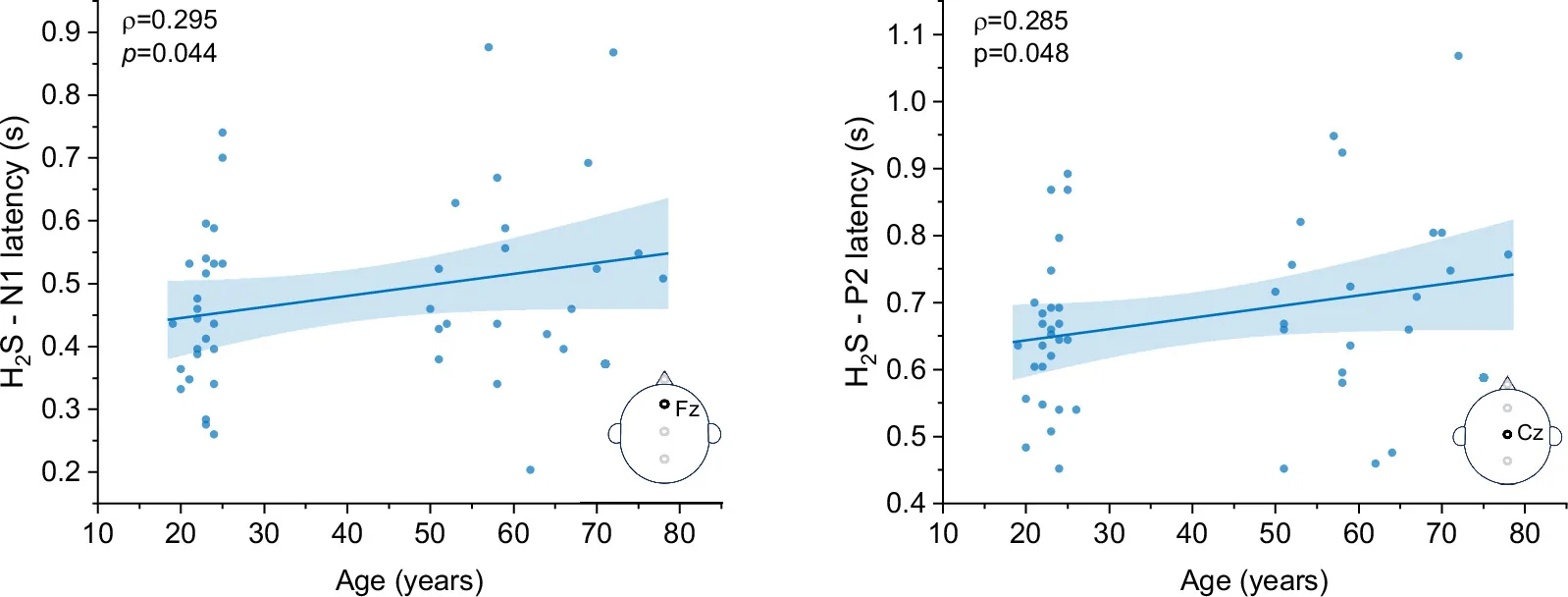
Significant correlations between age and CERP latencies in the H_2_S condition. Scatter plots visualize the relationships between age and CERP latencies, depicting individual data points, a linear fit (solid line), and 95% confidence intervals (shaded areas) to illustrate overall age-related trends. Spearman coefficients (*ρ*) are reported, as age was not normally distributed in the full sample. H_2_S hydrogen sulfide.

Participants in the old group exhibited significantly prolonged P2 latencies at Fz (young: 0.66 ± 0.14 s vs. old: 0.80 ± 0.35 s, *U* = 71, *p* = 0.033), Cz (young: 0.63 ± 0.14 s vs. old: 0.76 ± 0.22 s, *t* = 2.20, *p* = 0.035), and Pz (young: 0.64 ± 0.11 s vs. old: 0.76 ± 0.21 s, *t* = 2.18, *p* = 0.040) during CO_2_ perception (see Fig. 2). Correlation analyses further revealed significant associations between age and ERP latencies during H_2_S stimulation, with increasing age significantly correlated with prolonged N1 latency at Fz (*ρ* = 0.30, *p* = 0.044) and P2 latency at Cz (*ρ *= 0.29, *p* = 0.048) latencies (See Fig. 3). No significant differences were observed between age groups at the EOG electrode or under other odor conditions. Correlations between the time-domain measures and psychophysical olfactory scores are reported in Supplementary Result S1. Exploratory sex differences are reported in Supplementary Result S2.

### Electrophysiological: frequency results

**Fig. 4:**
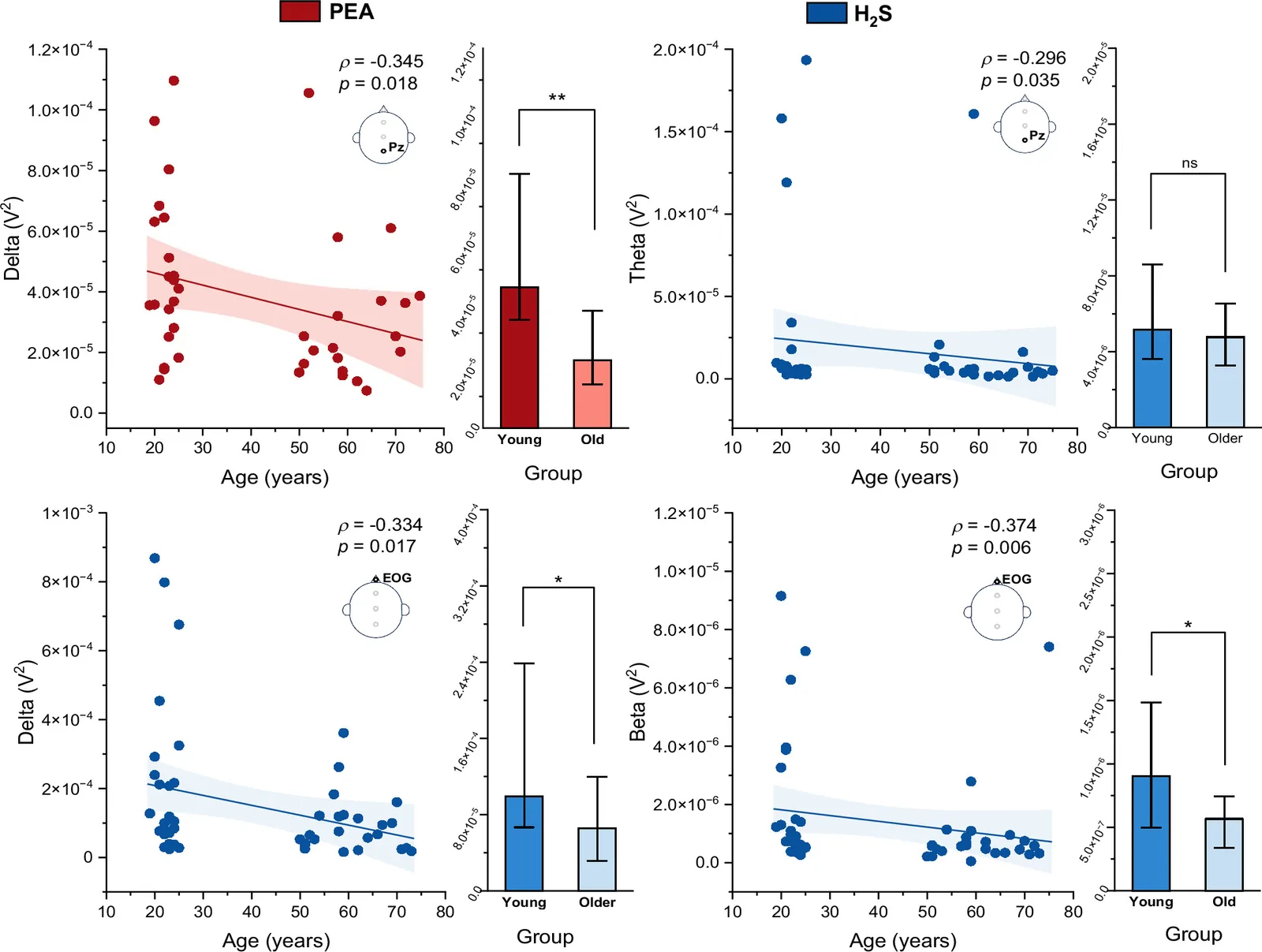
Significant relationships between age and EOG/CERP frequency power. Scatter plots visualize the correlations between age and spectral power for PEA (phenylethyl alcohol) and H_2_S (hydrogen sulfide), depicting individual data points, a linear fit (solid line), and 95% confidence intervals (shaded areas). Spearman correlation coefficients (*ρ*) are reported due to the non-normal distribution of the variables. Bar plots with error bars depict average (median) group-level responses and data variance (interquartile range). Group differences were evaluated using Mann–Whitney *U-*tests. EOG electro-olfactogram, ns non-significance; *p < 0.05; **p < 0.01.

As shown in Fig. 4, the young group exhibited significantly higher spectral power at the EOG or Pz electrodes across the frequency bands compared to the old group: (a) For the PEA condition, the young group showed significantly higher delta-band spectral power than the old group (4.44 × 10^−5^ V^2^ ± 5.16 × 10^−5^ V^2^ vs. 2.15 × 10^−5^ V^2^ ± 2.42 × 10^−5^ V^2^; *U* = 145, *p* = 0.006). (b) For the H₂S condition, the young group demonstrated significantly higher spectral power at the EOG electrode in the delta (9.93 × 10^−5^ V^2^ ± 17.22 × 10^−5^ V^2^ vs. 6.56 × 10^−5^ V^2^ ± 9.11 × 10^−5^ V^2^; *U* = 220, *p* = 0.049) and beta bands (9.04 × 10^−5^ V^2^ ± 9.86 × 10^−5^ V^2^ vs. 5.67 × 10^−5^ V^2^ ± 4.73 × 10^−5^ V^2^; *U* = 207, *p* = 0.017). No significant difference was observed for the CO_2_ condition.

Correlation analyses further revealed significant negative correlations between age and spectral power across frequency bands for both EOG and Pz signals in PEA and H_2_S conditions (*ρ *= -0.37 to -0.30, *p’s* < 0.05). See Fig. 4. Correlations between the frequency-domain measures and psychophysical olfactory scores are reported in Supplementary Result S1, and exploratory sex differences are reported in Supplementary Result S2.

## Discussion

The present study explored age-related olfactory alterations across psychophysical, peripheral, and central neural levels. In this healthy sample, age-related olfactory decline was evident across olfactory domains, manifested by significant differences in OT, OD, OI, and TDI scores between old and young groups, alongside significantly negative correlations between age and all these olfactory measures. As OT is particularly sensitive to peripheral encoding, while OD and OI involve higher-order cognitive processing and individual experience^15,36^, these psychophysical findings support the hypothesis that age-related decline involves multi-level mechanisms, consistent with earlier reports using the same Sniffin’ Sticks test^16^.

Regarding electrophysiological responses, time-domain findings indicated that aging primarily affects central processing speed. This was manifested by prolonged P2 latencies across all recorded sites during CO_2_ stimulation in the old group, and positive correlations between age and N1/P2 latencies for H_2_S. These findings align with previous CERP studies reporting age-related prolongation of P2 and/or N1 latencies for CO_2_^31^ and H_2_S^26,31^. Given that age-related changes in CERP latencies were not observed for PEA in the present study, one might attribute such an age effect to odor valence, as CO_2_ is perceived as a trigeminal unpleasant stimulus and H_2_S as a selectively unpleasant olfactory stimulus, whereas PEA is a selectively pleasant olfactory stimulus. However, studies at the perceptual level have shown that pleasant odors also show age-related declines in identification and discrimination, whereas responses to unpleasant odors may be relatively preserved^37,38^. Additionally, an EEG study^39^ reported age-related alteration at cortical beta-band activity during pleasant but not unpleasant stimulation. Thus, odor valence alone may not sufficiently explain the present pattern. Importantly, PEA latencies in the present study actually showed a positive trend with increasing age, although this did not reach significance (*ρ *= 0.24–0.31, *p* = 0.07–0.10). Therefore, the absence of significance for PEA does not necessarily indicate preserved central processing speed for pleasant odors with aging. It remains possible that overall low responses to PEA limited the sensitivity to detect subtle age-related latency changes. Future studies incorporating intensity and pleasantness ratings would be helpful to verify whether these null PEA effects directly relate to low perceptual response strength.

In terms of ERP components, our CERP results largely replicate previously reported age-related latency delays^24,28,31^. The P2 component, associated with higher-order cognitive evaluation and integrative processes^24,25,40^, appears to be particularly sensitive to aging, as evidenced by the robustly prolonged latencies observed in older individuals^24^. Although less common than P2^25,40^, N1 components, an earlier CERP component, has also been observed, showing age-related prolongation^26^, similar to the present finding.

Regarding CERP amplitudes, while several studies reported age-related reductions^28,31^, no significant age-related amplitude differences were observed in the present. This non-significance in amplitudes appears to align with other sensory modalities (e.g., visual), suggesting that latency may be a more sensitive index of age-related central sensory processing changes than amplitude^41^. Furthermore, no significant age-related differences were observed in EOG latencies, suggesting that age-related changes in neural processing speed may be more readily captured at the cortical than at the peripheral level. Importantly, this does not exclude age-related alteration at the mucosa level. On one hand, histological evidence from old individuals with a mean age of 74 years has demonstrated substantial age-related alterations, including a gradual loss of olfactory sensory neurons in the olfactory epithelium, which is accompanied by a reduction in glomeruli within the olfactory bulb^42^. On the other hand, this null finding should also be read with some caution, given the overall limited signal-to-noise ratios (SNR) of the chemosensory electrophysiological recordings. A relatively low SNR is well documented for cortical CERPs^43,44^, and the EOG has likewise been reported to be difficult to record, with a clear EOG not obtainable in ~30–40% of healthy individuals^29,45,46^. It is true that trigeminal (CO_2_) responses are generally easier to record and yield a higher SNR than responses to olfactory stimuli, at least at the cortical level^47^, which is why fewer trials were delivered for CO_2_ (i.e., 10) than for the other two stimuli (i.e., 20 each). Even so, the small number of artifact-free epochs ultimately available for CO_2_ (5.68 ± 2.03 epochs per participant, see Supplementary Table S4) may still challenge the reliability of peak/latency estimation to some degree, and potentially reduce the sensitivity to detect a group difference in CO_2_ EOG latency. This does not necessarily mean that the current findings are spurious. Precise stimulus timing is itself a determinant of SNR^44^, so the computer-controlled olfactometer with sharply defined onsets already constrains the jitter at issue^44^; waveform peak identification, in turn, relied not on literature-based time-windows alone but also on defined morphological criteria, with any ambiguous case judged by a second, experienced rater who was blinded to the age group (see Methods). Importantly, the number of artifact-free epochs entering the average did not differ between the young and old groups, so the null cannot be traced to selective epoch rejection. We therefore read this not as undermining the broader pattern of age-related effects, but as indicating that the peripheral latency warrants confirmation in studies with a larger number of trials.

Unlike time-domain results, frequency-domain analysis revealed age-related alterations at both peripheral and central olfactory encoding, indicated by decreased spectral power density across delta, theta and beta bands at Pz or EOG electrodes for PEA and H_2_S. Compared to the central level, age-related peripheral reduction appeared to be captured more sensitively in the frequency domain than through traditional time-domain measures. Given that EOG primarily reflects summed receptor potentials at the olfactory mucosa, reduced spectral power likely indicates diminished neural recruitment during odor processing in older adults. A recent study using intracranial electrophysiological recordings from the human piriform cortex reported that theta, beta, and gamma oscillations are involved in multiple olfactory tasks, such as odor detection and naming^48^. Additionally, delta oscillations have often been linked to respiration-related processes and anticipatory resetting prior to odor input^49,50^. The present findings of reduced oscillatory activity across multiple frequency bands with aging may suggest a broad decline in olfactory processing, affecting not only central cortical responses but also neural activity at the mucosa level. Furthermore, in contrast to the age-related slowing observed in the time domain, CO_2_ stimuli did not exhibit significant age-related alterations in neural oscillatory activity at either cortical or mucosal levels. This suggests that age-related oscillatory changes may be more sensitive to olfactory input than to trigeminal stimulation. The relative stability of the CO_2_ oscillatory response might tentatively reflect the defensive, warning function of the trigeminal system in detecting potentially harmful airborne irritants throughout the lifespan^51^, though this interpretation remains speculative and warrants further investigation, as an age-related slowing of cortical processing was still observed for this trigeminal stimulus. Across time- and frequency-domain analyses, the present results confirm that age-related processes are overall more pronounced in the olfactory system and less in the intranasal trigeminal system, consistent with some earlier works^52,53^.

It should be noted that the EOG and CERP were analyzed under two distinct time windows. This was because the two signals index physiologically distinct processes, each with its own characteristic timescale. The CERP is a fast response that largely resolves within ~1000 ms, whereas the EOG is a slow, sustained generator potential whose deflections of interest often occur around 1–2 s. Extending the cortical window to match the EOG window would likely introduce low-frequency drift, residual artifacts, and a reduced signal-to-noise ratio rather than an additional interpretable signal. The two signals were therefore not expected to align temporally, and our cross-level interpretation concerns how aging modulates each process rather than point-by-point correspondence between peripheral and cortical components. Accordingly, the absence of cortical data between 1500 and 4000 ms does not constrain the interpretation of age effects at either level.

Furthermore, exploratory correlation analyses (Supplementary Result S1) showed that stronger and faster electrophysiological responses, and greater oscillatory activities, were generally associated with better psychophysical olfactory performance. However, these correlations did not support a strict functional dichotomy (e.g., peripheral EOG reflecting OT, and central CERP reflecting OD/OI). Partial correlations controlling for age further showed that most time-domain associations persisted, most consistently the link between faster H_2_S P1 latency and better OD, suggesting that they were not simply age-driven. By contrast, nearly all frequency-domain associations were attenuated after controlling for age, suggesting they largely reflected age-related covariation. Given their exploratory nature, these correlations should be interpreted with caution.

Lastly, an exploratory analysis (Supplementary Result S2) of sex differences yielded results broadly consistent with previous reports of a female advantage in human olfactory function^31,54^, with higher OT scores, greater cortical and mucosal neural responses, faster peak latencies, and/or stronger neural oscillations in females than in males. These differences were largely observed in both the young and the old groups separately. Importantly, the young and old groups had comparable sex distribution, therefore the age-group differences reported in the main analyses are not confounded by a sex imbalance between groups.

This study has limitations regarding the number of odor trials. To minimize participant burden, we delivered 20 trials of PEA and H_2_S, and 10 trials of CO_2_. This, together with the overall low SNR discussed above, constrained the data available for analysis. Furthermore, both CERP and EOG are sensitive to ocular and muscle artifacts, with the EOG additionally sensitive to electrode placement and respiratory artifacts (the latter controlled here using a velopharyngeal closure technique)^27^. Because routine artifact rejection necessarily draws on an already small trial pool, a sufficient number of clean epochs could not always be retained for a reliable average, resulting in some missing cases (~15–38%) across conditions or recording sites in some participants after the exclusion of artifact-contaminated epochs. However, given our total sample size of 73, more than 40 samples were maintained per odor condition for evaluation. Future work should increase trial numbers to raise the signal-to-noise ratios and strengthen both the robustness of the findings and statistical power.

Another limitation is that cognitive status was not assessed. Thus, we cannot exclude subclinical cognitive decline in some older participants. This is relevant because the longer ERP latencies and reduced amplitudes have been linked to memory and general cognitive decline^55–57^, and reported in patients with mild cognitive impairment and Alzheimer’ disease^55,56^. Moreover, olfactory dysfunction is itself an early marker of cognitive decline and neurodegeneration^57,58^, so olfactory and cognitive aging are closely intertwined. Future studies incorporating cognitive assessment would help disentangle whether such age effects are olfactory-specific or partly reflect broader cognitive changes.

In summary, this study demonstrates a clear age-related alteration in olfactory and intranasal trigeminal processing at both behavioral, peripheral, and central neural levels. With aging, the abilities of olfactory sensitivity, discrimination, and identification decrease; neural oscillations across multiple frequency bands diminish at both the mucosal and scalp levels; and odor stimuli are processed more slowly at the higher central stage. These results confirm previous psychophysical and central nervous findings, while providing novel electrophysiological evidence for age-related changes at the mucosa level, together highlighting multi-level olfactory alterations with increasing age.

## Methods

### Participants

We recruited two age groups of participants: an older group (over 50 years) and a younger group (18–30 years). Inclusion criteria: (a) age ≥18 years, (b) being healthy, and (c) having an age-appropriated Sniffin’ Sticks TDI score (described in *“*Measurements*”* below). Exclusion criteria: (a) regularly smoking (>5 cigarettes/week), as current cigarette consumption is linked to olfactory dysfunction^59^, (b) history or current condition of nasal polyposis, chronic rhinosinusitis, or other sinonasal diseases, (c) neurodegenerative disease such as Parkinson’s disease or Alzheimer’s disease, (d) other significant health impairments. The study was conducted at the Smell and Taste Clinic, Department of Otorhinolaryngology, Technische Universität Dresden, Germany, with approval from the Ethics Committee at the Medical Faculty of TU Dresden, Germany (EK359082016). All participants provided written consent, and all procedures followed the Declaration of Helsinki.

### Procedures

**Fig. 5:**
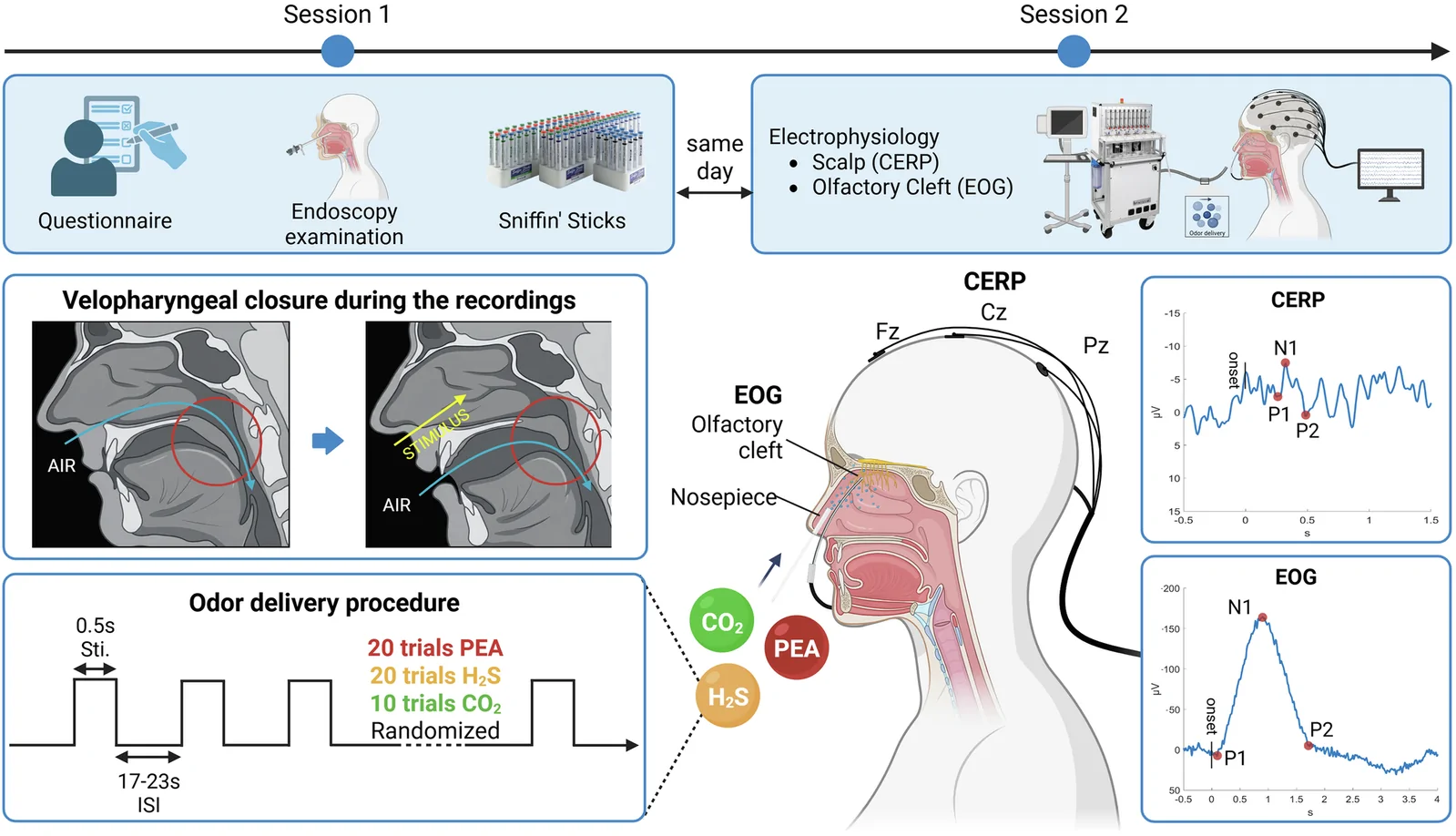
Study design and study procedure. PEA phenylethyl alcohol, H_2_S hydrogen sulfide, CO_2_ carbon dioxide, Sti. stimulus duration, ISI interstimulus interval, CERP chemosensory event-related potentials, EOG electro-olfactogram. This figure is created in BioRender (https://BioRender.com/80y61oo).

The study consisted of two sessions on the same day, totaling ~1.5 h (Fig. 5). The first session included a medical history interview, nasal endoscopy examination, and psychophysical olfactory testing. Eligible participants were trained to perform a breathing technique known as velopharyngeal closure to avoid nasal airflow and thereby minimize respiratory artifacts in EOG/EEG recordings^60^. In the second session, participants underwent electrophysiological recordings at the scalp (Fz, Pz, Cz) to obtain CERPs, and at the olfactory mucosa to obtain EOGs in response to chemosensory stimulation (details see below) via a computer-controlled olfactometer.

### Measurements

A short medical questionnaire was used to determine eligibility, including demographics, smoking history, sinonasal diseases, neurodegenerative diseases, and other health impairments^61^. Nasal endoscopy (Richard Wolf, Knittlingen, Germany; outer diameter 1.9 mm) was used to rule out nasal polyps and any other sinunasal diseases^62^.

The Sniffin’ Sticks test battery (Burghart, Holm, Germany) was used to assess olfactory function, comprising odor threshold (OT), discrimination (OD) and identification (OI) tests. Each subtest yields a maximum score of 16, and the sum reported as the total TDI score (ranged 1–48)^15,36^. Higher TDI scores indicate better overall olfactory function. Eligibility required a TDI score equal to or higher than the age-specific cutoff according to the latest normative data^16^.

Regarding the EOG and CERP recordings, a total of ten electrodes connected to an eight-channel EEG amplifier (Burghart, Holm, Germany) were used for EOG/CERP recordings. EOG was recorded by a Ringer-agar (1%) filled tubular electrode containing a chlorided silver wire (0.3 mm diameter of the wire, 0.4 mm inner and 0.8 mm outer diameter of the tubing)^60^. The electrode was placed under endoscopic control (Richard Wolf, Knittlingen, Germany; outer diameter 1.9 mm) in the nasal cavity at the olfactory epithelium atop the middle turbinate^46^. The electrode was stabilized using a clip mounted on a frame similar to lensless glasses^27^. CERP was recorded at Fz, Cz, and Pz according to the standard 10/20 system, with eye-blink artifacts being monitored at the Fp2. Reference electrodes were placed on the earlobes (A1 + A2). Two mastoid electrodes served as ground. A total of 50 stimuli, including 20 phenylethyl alcohol (PEA), 20 hydrogen sulfide (H_2_S) and 10 carbon dioxide (CO_2_) stimuli, were presented in a block-randomized sequence via a computer-controlled olfactometer. Each stimulation lasted 500 ms, with an interstimulus interval of 17–23 s to avoid habituation^63^. Recording epochs (125 Hz sampling rate) began 0.5 s before onset and lasted 7.192 s after the stimulus offset. To mask the olfactometer’s switching sounds, the participants wore headphones with white noise (~75 dB SPL). To maintain attention, participants performed a simple tracking task, using a mouse to follow a moving square displayed on the screen. Total recording duration was ~20 min.

Two olfactory stimuli and one trigeminal stimulus were used for EOG/CERP recordings. Specifically, (1) phenylethyl alcohol (PEA, CAS 60-12-8, Sigma-Aldrich Chemistry, Steinheim, Germany, 40% v/v), a pleasant, rose-like olfactory stimulus; (2) hydrogen sulfide (H_2_S, Air Liquide, Ottendorf-Okrilla, Germany; 5 ppm), an unpleasant rotten-egg olfactory stimulus; (3) carbon dioxide (CO_2_, Air Liquide, Ottendorf-Okrilla, Germany; 50% v/v), an odorless smell for almost exclusively trigeminal stimulation. Stimuli were presented using a computer-controlled olfactometer (OM6b, Burghart-Messtechnik, Holm, Germany) at 36 °C and 80% humidity to resemble nasal conditions. A constant flow rate of 6 L/min was maintained through Teflon™ tubing (8 cm length and 2 mm inner diameter).

### EEG preprocessing

Offline signal processing and spectral analysis were performed using Letswave 7 (https://letswave.cn/) and custom scripts written in MATLAB (The MathWorks, Inc., Natick, MA, USA). The continuous EEG and EOG data, sampled at 125 Hz, were segmented into epochs time-locked to the onset of the odor stimulation. Analysis was restricted to three midline cortical electrodes (Fz, Cz, and Pz) to assess central nervous system processing, and one nasal channel (EOG) to evaluate peripheral olfactory mucosa responses. The Fp2 electrode and other auxiliary channels were excluded from the final spectral analysis to minimize ocular artifacts and focus on specific regions of interest. To account for the different temporal dynamics of cortical and peripheral olfactory responses, two distinct time windows were defined relative to the stimulus onset: (1) Cortical response window: For Fz, Cz, and Pz channels, data were analyzed over the 0 to 1500 ms post-stimulus interval to capture early cognitive processing. This window encompasses the CERP components of interest, which are complete before 1500 ms, consistent with the windows widely used in previous studies^64–66^. (2) Peripheral response window: For the EOG channel, a broader time window of 0 to 4000 ms was utilized to encompass the prolonged depolarization of the olfactory receptor neurons. The EOG is a slow potential whose latest deflection of interest (i.e., EOG P2) can occur as late as ~4 s^23^. This wider window was therefore applied to capture the complete peripheral response. For time-domain analysis, a low-pass filter at 15 Hz was applied, with baseline correction performed using the -500 to 0 ms interval. All epochs were visually inspected to identify and reject those containing artifacts like eye-blinks, muscular artifacts, or movement-related artifacts. Artifact-free epochs were then averaged per odor condition for each participant, if at least three artifact-free epochs were available for averaging^67^. Amplitudes and latencies of EOG (P1, N1, P2) and CERP (P1, N1, P2 at Fz, Cz, and Pz) components were identified manually via visual inspection of each participant’s averaged waveform for each condition. Approximate time-windows for peak detection were based on previous chemosensory electrophysiology studies (for CERP, ~250–320 ms for P1, 320–450 ms for N1, and 530–800 ms for P2^28^; for the EOG, ~100–500 ms for P1, 1000–2000ms for N1, and 1500–4000 ms for P2^23,46,68,69^) and served as a reference rather than as strict fixed windows, because chemosensory peak latencies are varied across individuals. Additionally, two features typical of chemosensory ERPs were used to support identification and to distinguish genuine components from noise in low-SNR waveforms: (1) the deflection appeared at a largely consistent latency across Fz, Cz, and Pz traces; (2) its amplitude generally followed a scalp distribution, being larger at Pz and Cz and gradually decreasing toward Fz^24,28,43^. Because the EOG was recorded from a single channel at the olfactory mucosa, these cross-site features could not be applied to it. EOG peaks were therefore identified primarily from waveform morphology and the literature-based time windows mentioned above, and EOG latency estimates were accordingly less constrained than the cortical ERP. All peaks were first marked by one author (Y.M.) and then reviewed together with a second experienced rater (T.H.), who was blinded to participants’ age group. Ambiguous cases were examined jointly and resolved by consensus. When a component was absent or too ambiguous to be resolved by consensus, that specific peak was recorded as a missing value for the corresponding participant, condition, and channel, while the remaining identifiable components from the same waveform were retained. For frequency analysis, a band-pass filter of 0.5–30 Hz was applied, and the segmented time-domain signals for each trial and channel were transformed into the frequency domain using the fast Fourier transform (FFT). The power spectral density (PSD) was computed, and the spectral power (V^2^) was extracted by integrating the power spectrum within four specific frequency bands defined as follows: 0.5–3.5 Hz (Delta), 4–7 Hz (Theta), 8–12 Hz (Alpha), 13–28 Hz (Beta). Gamma activity was not analyzed because the 125 Hz sampling rate and its inherent anti-aliasing hardware filter severely attenuate high-frequency signals, rendering such estimations unreliable. Single-trial spectral power values were grouped according to the odorant class, and the mean spectral power for each frequency band and channel was calculated for each subject and odor condition.

### Statistical analysis

Data were analyzed using SPSS 30.0 software (IBM Corp., Armonk, NY, USA). Continuous variables, including age, BMI, OT, OD, OI, TDI, ERP/EOG measures, and the number of accepted ERP/EOG epochs, were presented as the mean and standard deviation if normally distributed (confirmed via the Shapiro–Wilk test), or as the median and interquartile range (IQR) if non-normally distributed. Categorical variables, including sex and smoking status, were shown as counts and percentages. For group comparisons (young vs. old), data distribution was evaluated within each age group separately. Continuous variables were compared using independent-samples *t*-tests when both age groups met the normality assumption, or Mann–Whitney *U-*tests when they did not. Categorical variables were compared using the chi-square test (χ^2^). For correlation analyses examining the relationships between age, psychophysical scores, and electrophysiological responses, Pearson correlation (coefficients reported as *r*) was used when both variables in a pair were normally distributed across the whole sample. If at least one variable in a pair was non-normally distributed, Spearman correlation (coefficient reported as *ρ*) were employed. The level of significance was set at alpha = 0.05.

## Supplementary information

Supplement R1

## Data Availability

The datasets generated and/or analyzed during the current study are not publicly available due to ethical restrictions and data protection requirements governing sensitive participant health information, and because participants were explicitly assured during the informed consent process that their data would not be shared with or disclosed to third parties. However, a de-identified minimal dataset is available from the corresponding author on reasonable request, subject to a formal Data Use Agreement (DUA) and approval by the local Institutional Review Board.
