# Supplementary material for: Age-related changes in olfactory function: electrophysiological recordings from the olfactory mucosa and human brain
Source: NPJ Aging. 2026 Sep 17;12(1):130. doi: 10.1038/s41514-026-00478-3 (PMC13586338; doi:10.1038/s41514-026-00478-3)
Supplement: Supplementary file 1 [file 41514_2026_478_MOESM1_ESM.docx]

**Supplement**

**Table S1. Number of artifact-free epochs for averaging – CERP - Whole sample**

|  | Sample size | Number of artifact-free epochs | | | | | |
| --- | --- | --- | --- | --- | --- | --- | --- |
|  |  | Total | Range | Median | IQR | Mean | SD |
| PEA | 62 | 586 | 3-19 | 9.50 | 7.25 | 9.45 | 4.40 |
| H_2_S | 62 | 581 | 3-20 | 9.00 | 7.25 | 9.37 | 4.32 |
| CO_2_ | 45 | 240 | 3-10 | 5.00 | 2.50 | 5.33 | 1.81 |

**Table S2. Number of artifact-free epochs for averaging – CERP – Young group**

|  | Sample size | Number of artifact-free epochs | | | | | |
| --- | --- | --- | --- | --- | --- | --- | --- |
|  |  | Total | Range | Median | IQR | Mean | SD |
| PEA | 34 | 330 | 3-18 | 10.00 | 5.00 | 9.71 | 3.79 |
| H_2_S | 34 | 331 | 3-20 | 9.00 | 7.00 | 9.74 | 4.45 |
| CO_2_ | 23 | 115 | 3-9 | 5.00 | 2.00 | 5.00 | 1.65 |

**Table S3. Number of artifact-free epochs for averaging – CERP – Old group**

|  | Sample size | Number of artifact-free epochs | | | | | |
| --- | --- | --- | --- | --- | --- | --- | --- |
|  |  | Total | Range | Median | IQR | Mean | SD |
| PEA | 28 | 256 | 3-19 | 7.50 | 8.75 | 9.14 | 5.10 |
| H_2_S | 28 | 250 | 3-19 | 9.00 | 5.75 | 8.93 | 4.19 |
| CO_2_ | 22 | 125 | 3-10 | 6.00 | 3.00 | 5.68 | 1.94 |

Note. Sample size=number of participants with at least three artifact-free epochs available for ERP averaging; Total=total number of artifact-free epochs per odor conditions; Range= range of artifact-free epochs; IQR=interquartile range; SD=standard deviation; CERP= chemosensory event-related potential.

The number of artifact-free epochs used for ERP averaging did not differ significantly between the young and the old groups in any odor conditions (*t*=0.48-1.27, p’s=0.21-0.63).

**Table S4. Number of artifact-free epochs for averaging – EOG - Whole sample**

|  | Sample size | Number of artifact-free epochs | | | | | |
| --- | --- | --- | --- | --- | --- | --- | --- |
|  |  | Total | Range | Median | IQR | Mean | SD |
| PEA | 59 | 509 | 3-19 | 9.00 | 7.00 | 8.63 | 4.28 |
| H_2_S | 57 | 492 | 3-14 | 9.00 | 8.00 | 8.63 | 3.75 |
| CO_2_ | 47 | 267 | 3-10 | 6.00 | 3.00 | 5.68 | 2.03 |

**Table S5. Number of artifact-free epochs for averaging – EOG – Young group**

|  | Sample size | Number of artifact-free epochs | | | | | |
| --- | --- | --- | --- | --- | --- | --- | --- |
|  |  | Total | Range | Median | IQR | Mean | SD |
| PEA | 30 | 266 | 3-16 | 9.00 | 5.50 | 8.87 | 3.36 |
| H_2_S | 32 | 283 | 3-14 | 9.00 | 7.75 | 8.84 | 3.79 |
| CO_2_ | 27 | 160 | 3-10 | 6.00 | 3.00 | 5.93 | 2.04 |

**Table S6. Number of artifact-free epochs for averaging – EOG – Old group**

|  | Sample size | Number of artifact-free epochs | | | | | |
| --- | --- | --- | --- | --- | --- | --- | --- |
|  |  | Total | Range | Median | IQR | Mean | SD |
| PEA | 29 | 243 | 3-19 | 7.00 | 9.00 | 8.38 | 5.10 |
| H_2_S | 25 | 209 | 3-14 | 9.00 | 8.00 | 8.36 | 3.75 |
| CO_2_ | 20 | 107 | 3-10 | 5.00 | 3.75 | 5.35 | 2.03 |

Note. Sample size=number of participants with at least three artifact-free epochs available for ERP averaging; Total=total number of artifact-free epochs per odor conditions; Range= range of artifact-free epochs; IQR=interquartile range; SD=standard deviation; CERP= chemosensory event-related potential.

The number of artifact-free epochs used for EOG averaging did not differ significantly between the young and the old groups in any odor conditions (*t*=0.43-0.96, p’s=0.34-0.67).

**Result S1. Correlations between psychophysical olfactory scores and CERP/EOG measures**

Correlations relationships were examined using the *Pearson* (*r*) or *Spearman* (*ρ*) correlations, depending on the data distribution. To test whether these brain-behavior relationships hold independently of age, we additionally computed partial correlations (*r*) controlling for age.

(1) **Correlation analyses**

**Time-domain measures:**

a) TDI significantly correlated with PEA Fz N1 amplitude (*r*=-0.33, *p*=0.027), H_2_S Fz P1 latency (*r*=-0.41, *p*=0.029), H_2_S Pz P1 latency (*r*=-0.38, *p*=0.041), CO_2_ Pz P1 amplitude (r=0.49, p=0.032), and CO_2_ EOG P1 amplitude (ρ=0.40, *p*=0.034): better overall olfactory function was associated with greater neural responses to PEA and CO_2_ and with faster processing speed for H_2_S.
b) OT correlated with PEA Pz P2 amplitude (*r*=0.31, *p*=0.036), CO_2_ Pz P1 amplitude (*r*=0.53, *p*=0.019), and CO_2_ Cz N1 amplitude (*r*=-0.35, *p*=0.046): greater olfactory sensitivity was associated with larger CERP responses to PEA and CO_2_.
c) OD correlated with PEA Fz N1 amplitude (*ρ*=-0.32, *p*=0.035), PEA Pz N1 amplitude (*ρ*=-0.34, *p*=0.018), H_2_S Fz P1 latency (*ρ*=-0.58, *p*=0.001), H_2_S Cz P1 latency (*ρ*=-0.55, *p*=0.002), H_2_S Pz P1 latency (*ρ*=-0.49, *p*=0.007), H_2_S Cz N1 latency (*ρ*=-0.31, *p*=0.031), H_2_S Cz N1 amplitude (*ρ*=-0.47, *p*=0.001), and CO_2_ Cz P2 amplitude (*ρ*=0.39, *p*=0.021): better discrimination was associated with larger neural response and faster processing speed.
d) OI correlated with CO_2_ Cz P1 amplitude (*ρ*=0.50, *p*=0.024), CO_2_ EOG P1 amplitude (*ρ*=0.42, *p*=0.025), CO_2_ Cz P2 latency (*ρ*=-0.26, *p*=0.034), and CO_2_ Pz P2 latency (*ρ*=-0.36, *p*=0.039): better olfactory identification was associated with larger and faster neural responses to CO_2_.

**Frequency-domain measures:**

e) TDI correlated with PEA Pz delta (*ρ*=0.29, p=0.046), H_2_S Cz theta (*ρ*=0.28, p=0.048), H_2_S Pz theta (*ρ*=0.29, p=0.039), CO_2_ Fz theta (*ρ*=0.34, p=0.014), CO_2_ Cz theta (*ρ*=0.31, p=0.027): better overall olfactory function was associated with higher cortical theta power across odors.
f) OD correlated with PEA Pz delta (*ρ*=0.39, p=0.006), H_2_S EOG theta (*ρ*=0.29, p=0.040), H_2_S EOG beta (*ρ*=0.32, p=0.019): better discrimination was associated with greater neural oscillatory activity to olfactory stimulus at both cortical and mucosal levels.
g) OI correlated with H_2_S Pz theta (*ρ*=0.28, p=0.048), CO_2_ Fz theta (*ρ*=0.35, p=0.009), CO_2_ Pz delta (*ρ*=0.30, p=0.039), and CO_2_ Pz theta (*ρ*=0.29, p=0.040): better identification was associated with greater oscillatory activity, mainly in the theta band, for the two unpleasant odors at cortical level.
(2) **Partial correlations controlling for age**

**Time-domain measures:**

a) TDI remained correlated with PEA Fz N1 amplitude (*r*=-0.36, *p*=0.015), H_2_S Fz P1 latency (*r*=-0.45, *p*=0.017), H_2_S Cz P1 latency (*r*=-0.42, *p*=0.022), H_2_S Pz P1 latency (*r*=-0.41, *p*=0.030) and CO_2_ Cz P1 amplitude (*r*=0.51, *p*=0.024)
b) OT was correlated only with CO_2_ Pz P1 amplitude (*r*=0.51, *p*=0.032).
c) OD remained correlated with PEA Pz P1 amplitude (*r*=-0.38, *p*=0.045), PEA Fz N1 amplitude (*r*=-0.35, *p*=0.018), PEA Pz N1 amplitude (*r*=-0.39, *p*=0.008), H_2_S Fz P1 latency (*r*=-0.65, *p*<0.001), H_2_S Cz P1 latency (*r*=-0.66, *p*<0.001), H_2_S Pz P1 latency (*r*=-0.59, *p*=0.001), H_2_S Cz N1 amplitude (*r*=-0.42, *p*=0.005), and CO_2_ Cz P2 amplitude (*r*=0.45, *p*=0.009)
d) OI was correlated with PEA EOG P2 latency (*r*=0.35, *p*=0.030), CO_2_ Cz P1 amplitude (*r*=0.52, *p*=0.022)

**Frequency-domain measures:**

a) Only the association between OI and CO_2_ EOG theta power remained significant (*r*=0.30, *p*=0.033). None of the other frequency-domain correlations survived adjustment for age.

**Result S2. Gender effects on olfaction**

Gender differences were examined using the independent-samples *t*-test or Mann-Whitney *U* test, depending on the data distribution. Significant gender effects were found in both the young and old groups, with females generally showing greater olfactory responses than males across multiple measures.

(1) **Psychophysical measures**

- Across the whole sample, females had higher OT scores than males (8.63±2.40 vs. 7.48±2.11; *t*=2.03, *p*=0.045). This difference was not significant when the young and old groups were analyzed separately (all *p’s*>0.05).
- OD, OI, and TDI showed no sex differences, in neither the whole sample nor the young or old groups (all *p’s*>0.05).

(2) **Time-domain measures**

- **Whole sample**: Females showed greater PEA Pz P2 (4.53±2.43 vs. 2.70±2.06 μV; *t*=2.63, *p*=0.006) and Cz P2 amplitudes (4.18±1.81 vs. 1.40±2.95 μV; *U*=109, *p*<0.001); greater H_2_S Cz N1 amplitude (-4.32±2.33 vs. -2.26±2.10 μV; *t*=2.92, *p*=0.006); and greater CO_2_ Cz P2 amplitude (6.55±4.55 vs. 3.61±2.31 μV; *t*=2.28, *p*=0.021), together with a longer Pz N1 latency (0.51±0.16 vs. 0.40±0.14 s; *t*=2.05, *p*=0.049). In contrast, females showed a smaller (less negative) CO_2_ Cz N1 amplitude than males (-2.31±2.19 vs. -3.95±2.30 μV; *t*=2.12, *p*=0.042).
- **Young group**: Females showed greater PEA Pz P2 (3.62±2.64 vs. 1.76±2.42 μV; *t*=2.45, *p*=0.021) and Cz P2 amplitudes (4.24±2.23 vs. 1.99±3.24 μV; *U*=58.0, p=0.045); greater H_2_S Cz N1 amplitude (-5.13±2.30 vs. -2.49±2.38 μV; *t*=2.76, *p*=0.011); and shorter H_2_S EOG N1 (0.78±0.36 vs. 1.09±0.38 s; *t*=2.10, *p*=0.047) and P2 latencies (1.28±0.50 vs. 1.71±0.42 s; *t*=2.29, *p*=0.032).
- **Old group**: Females showed greater PEA Pz N1 (-3.36±1.52 vs. -1.15±1.41 μV; *t*=3.08, *p*=0.007) and Cz P2 amplitudes (4.07±0.81 vs. 1.16±2.21 μV; *t*=3.69, *p*=0.002), and greater H_2_S EOG N1 amplitude (-8.73±11.94 vs. -4.46±4.27 μV; *U*=26, *p*=0.043).

(3) **Frequency-domain measures (all power values in V^2^)**

- **Whole sample**: Females showed greater Cz alpha power than males for PEA (2.92×10^-6^±1.92×10^-6^ vs. 1.98×10^-6^±1.40×10^-6^; *U*=203, *p*=0.039), H_2_S (2.81×10^-6^±2.25×10^-6^ vs. 1.62×10^-6^±1.59×10^-6^; *U*=184, *p*=0.007), and CO_2_ (2.53×10^-6^±3.41×10^-6^ vs. 1.66×10^-6^±1.43×10^-6^; *U*=198, *p*=0.015). For H_2_S, females additionally showed greater Cz theta (6.19×10^-6^±4.71×10^-6^ vs. 5.16×10^-6^±3.78×10^-6^; *U*=196, *p*=0.026) and Cz beta power (1.02×10^-6^±1.03×10^-6^ vs. 0.63×10^-6^±0.53×10^-6^; *U*=215, *p*=0.035). Females also showed greater Pz alpha power for H_2_S (2.82×10^-6^±3.09×10^-6^ vs. 1.62×10^-6^±1.58×10^-6^; *U*=215, *p*=0.035) and CO_2_ (3.41×10^-6^±3.47×10^-6^ vs. 1.74×10^-6^±2.34×10^-6^; *U*=187, *p*=0.009).
- **Young group**: For H_2_S, females showed greater Cz alpha power (2.81×10^-6^±2.60×10^-6^ vs. 1.67×10^-6^±1.41×10^-6^; *U*=46, *p*=0.035), and greater EOG delta (2.07×10^-4^±3.14×10^-4^ vs. 0.75×10^-4^±0.98×10^-4^; *U*=49, *p*=0.020) and EOG beta power (1.40×10^-6^±3.32×10^-6^ vs. 0.63×10^-6^±5.63×10^-6^; *U*=45, *p*=0.046).
- **Old group**: For H_2_S, females showed greater Cz theta power (8.46×10^-6^±12.52×10^-6^ vs. 4.71×10^-6^±4.38×10^-6^; *U*=31, *p*=0.045). For CO_2_, females showed greater Pz power across delta (3.68×10^-5^±7.11×10^-5^ vs. 1.75×10^-5^±1.98×10^-5^; *U*=29, *p*=0.033), theta (6.84×10^-6^±13.40×10^-6^ vs. 3.85×10^-6^±3.68×10^-6^; *U*=30, *p*=0.017), alpha (5.33×10^-6^±4.85×10^-6^ vs. 1.74×10^-6^±1.88×10^-6^; *U*=21, *p*=0.004), and beta bands (1.54×10^-6^±1.92×10^-6^ vs. 0.84×10^-6^±0.69×10^-6^; *U*=27, *p*=0.010).
